# The mechanism and role of intracellular α-ketoglutarate reduction in hepatic stellate cell activation

**DOI:** 10.1042/BSR20193385

**Published:** 2020-03-12

**Authors:** Jianjian Zhao, Yueping Jiang, Xueguo Sun, Xishuang Liu, Fuguo Liu, Mingquan Song, Lingyun Zhang

**Affiliations:** Department of Gastroenterology, the Affiliated Hospital of Qingdao University, China

**Keywords:** α-ketoglutarate, hepatic stellate cells, isocitrate dehydrogenase 2, metabolite

## Abstract

**Background:** The activation of hepatic stellate cells (HSCs) plays a central role in liver fibrosis. α-ketoglutarate is a natural metabolite and previous studies have shown that increase in intracellular α-ketoglutarate can inhibit HSC activation. **Aim:** The aim of the present study is to determine the changes and role of intracellular α-ketoglutarate in HSC activation and clarify its mechanism of action. **Methods:** A human HSC cell line (LX-2) and the primary mouse HSC were used in the present study. We detected the changes of intracellular α-ketoglutarate levels and the expression of enzymes involved in the metabolic processes during HSC activation. We used siRNA to determine the role of intracellular α-ketoglutarate in HSC activation and elucidate the mechanism of the metabolic changes. **Results:** Our results demonstrated that intracellular α-ketoglutarate levels decreased with an HSC cell line and primary mouse HSC activation, as well as the expression of isocitrate dehydrogenase 2 (IDH2), an enzyme that catalyzes the production of α-ketoglutarate. In addition, knockdown of IDH2 efficiently promoted the activation of HSCs, which was able to be reversed by introduction of an α-ketoglutarate analogue. Furthermore, we demonstrated that α-ketoglutarate regulated HSC activation is independent of transforming growth factor-β1 (TGF-β1). **Conclusions:** Our findings demonstrated that decrease in IDH2 expression limits the production of α-ketoglutarate during HSC activation and in turn promotes the activation of HSCs through a TGF-β1 independent pathway. The present study suggests that IDH2 and α-ketoglutarate may be potential new targets for the prevention and treatment of liver fibrosis.

## Introduction

Liver fibrosis results from many chronic liver diseases, including viral hepatitis, autoimmune hepatitis, alcoholic liver disease, and non-alcoholic steatohepatitis. Ultimately, chronic inflammation despite the underlying cause may lead to liver cirrhosis, liver failure, and even hepatocellular carcinoma [1]. It is currently unclear the mechanisms of liver fibrosis. Identifying and understanding these mechanisms would facilitate the development of preventive and therapeutic approaches for cirrhosis and hepatocellular carcinoma. Activation or transdifferentiation of hepatic stellate cells (HSCs) from a quiescent state into a myofibroblast-like phenotype plays a central role in liver fibrosis. Etiological treatments such as antiviral drugs that remove the underlying cause of viral-induced liver injury may halt the process of liver fibrosis. Unfortunately, not all these pathogenic factors can be eliminated [2–4]. Moreover, after cessation of the fibrotic inducing stimulus, about 50% of the activated HSCs survive in a seemingly quiescent state and are primed to quickly reactivate in response to pro-fibrotic stimuli [5,6]. Thus, direct regulation of HSC activation has been the focus of the present study [7].

In previous studies, various signaling molecules involved in HSC activation have been identified, such as transforming growth factor-β1 (TGF-β1) [8]. However, no antifibrotic therapy has been approved to date, largely due to tolerability issues. In recent years, increasing evidence suggests that metabolites can affect cell phenotype transdifferentiation [9–11]. Several studies suggest that some metabolites such as fatty acid, glucose, cholesterol, and amino acids can regulate HSC activation and even liver fibrosis. It is thought that regulation of metabolites may be a safer target in comparison with the regulation of signal transduction events [12–14].

α-Ketoglutarate is a natural metabolite. Our previous studies have shown that direct increase in intracellular α-ketoglutarate can dramatically inhibit HSC cell line activation *in vitro* and attenuate CCl_4_-induced liver fibrosis *in vivo*. Moreover, increase in intracellular α-ketoglutarate does not exacerbate hepatocyte injury [15,16].

In light of these findings, we speculate that intracellular α-ketoglutarate may decrease with HSC activation and a lack of α-ketoglutarate may promote the activation of HSCs. We aimed to determine the role of intracellular α-ketoglutarate in HSC activation and elucidate the mechanism of the metabolic changes. The present study may suggest new targets for the prevention and treatment of liver fibrosis.

## Materials and methods

### Primary mouse HSC isolation, cell culture, treatment, and transfection

All procedures were performed in the Affiliated Hospital of Qingdao University according to protocols approved by the Ethics Committee from the Affiliated Hospital of Qingdao University. Mice were killed by decapitation after intraperitoneal anesthesia with chloral hydrate (0.25 ml of 10% solution per 100 g of body weight). Primary mouse HSCs were isolated from mice using a two-step collagenase-pronase perfusion technique of mouse livers as previously described [17]. Culture purity, assessed by immunohistochemistry for desmin, was > 95%. Human activated HSC cell line, LX-2 was obtained from Procell Life Science & Technology (Procell, China). The primary mouse HSCs and the LX-2 cells were cultured in Dulbecco’s Modified Eagle Medium (DMEM, Gibco, U.S.A.) supplemented with 10% fetal bovine serum (FBS, Gibco, U.S.A.), 2 mmol/l L-glutamine, 100 U/ml penicillin and 100 μg/ml streptomycin (Gibco, U.S.A.) at 37°C and with 5% CO_2_. Specific predesigned small interfering RNA (siRNAs) (sense, CAGUAUGCCAUCCAGAAGA, and antisense, UCUUCUGGAUGGCAUACUG) (Genepharma, China) were used to silence isocitrate dehydrogenase 2 (IDH2) expression. IDH2 siRNA (final concentration of 100 nM) were transfected into cells using Lipofectamine 2000 reagent (Invitrogen, U.S.A.) following the manufacturer’s instructions. Transfection efficiency was evaluated by Western blot analysis 72 h after transfection. The non-targeted siRNA was used as a negative control. Cells were seeded on tissue culture plates and left overnight to attach before stimulation with dimethyl α-ketoglutarate (DMKG; TCI, Japan; 4 mM) or TGF-β1 (MCE, USA; 5 ng/ml) for 24 h.

### α-Ketoglutarate assays

The assay was performed using the α-Ketoglutarate Assay Kit according to the manufacturer’s instructions (MAK054, Sigma Aldrich, U.S.A.). In brief, intracellular α-ketoglutarate levels were determined by a coupled enzyme assay, which results in a colorimetric (570 nm) product, proportional to the levels of α-Ketoglutarate present.

### Quantitative real-time PCR

Total RNA was isolated from cells using Trizol (Takara, Japan). All RNA samples were treated with gDNA Eraser (Takara, Japan) to remove residual genomic DNA contamination before performing reverse transcription of total RNA. Reverse transcription of total RNA to cDNA was performed using Prime Script RT reagent Kit (Takara, Japan) according to the manufacturer’s protocol. Real-time reverse transcription PCR analysis was done with the Light Cycler 2.0 System (Roche, Germany) in 10 μl reactions containing 1 μl of cDNA, 1 μl of each forward and reverse primers (10 μmol/l), 5 μl SYBR Green 1 Master Mix (Takara, Japan), and 2 μl RNase-free H_2_O. The reactions were carried out with the following cycling variables: an initial denaturation of 10 min at 95°C, then amplified for 40 cycles (30 s at 95°C, 30 s at 60°C) and followed by a final extension step. The relative amount of mRNA levels was calculated by 2^−ΔΔCT^ method. Relative gene expression was normalized to GAPDH expression. The primers used for PCR amplification were as follows: α-SMA (human): Forward 5′-CTT GAG AAG AGT TAC GAG TTG-3′ and Reverse 5′-GAT GCT GTT GTA GGT GGT T-3′, α-SMA (mouse): Forward 5′-AGA ACA CGG CAT CAT CAC-3′ and Reverse 5′-TCC AGA GTC CAG CAC AAT-3′, collagen I α1 (Col1α1, human): Forward 5′-GTG ACG AGA CCA AGA ACT-3′ and Reverse 5′-CTC ATC ATA GCC ATA AGA CAG-3′, Col1α1(mouse): Forward 5′-GTG GCG GTT ATG ACT TCA-3′ and Reverse 5′-CTG CGG ATG TTC TCA ATC T-3′, IDH1 (human): Forward 5′-CCT GGG CCT GGA AAA GTA GA-3′ and Reverse 5′-CCA ACC CTT AGA CAG AGC CA-3′, IDH2 (human): Forward 5′-TCG GGT GGC TTT GTG TGG-3′ and Reverse 5′-ATG CTG GCG ATG GGG TTG G-3′, IDH2 (mouse): Forward 5′-GAC AAG CAC TAT AAG ACT GAC-3′ and Reverse 5′-TCT GGT GTT CTC GGT AAT G-3′, branched-chain aminotransferase 1 (BCAT1, human): Forward 5′-AAG AAG CCT ACC AAA GCC CT-3′ and Reverse 5′-GCC GTA ATT CCC TCC CAT CT-3′, oxoglutarate dehydrogenase (OGDH, human): Forward 5′-CCA TTC CCC TTT GAC CTC CT-3′ and Reverse 5′-CAG GTG GGT CTT CTT GTT GC-3′, glutamate dehydrogenase 1 (GDH, human): Forward 5′-TAT TGA CCC AAA GGA ACT G-3′ and Reverse 5′-TCC TCC AGC ATT CAA GTA G-3′, solute carrier family 1 member 4 (SLC1A4, human): Forward 5′-CTG GCT GTG GAC TGG ATT GTG-3′ and Reverse 5′-TCC TGC TCG CCT TTC TTT GTT-3′, solute carrier family 1 member 5 (SLC1A5, human): Forward 5′-GGA GAT GGA GGA TGT GGG TTT A-3′ and Reverse 5′-AGG AAG CGG TAG GGG TTT TT-3′, glutamate-ammonia ligase (GLUL, human): Forward 5′-ATT ACT GTG GTG TGG GAG CA-3′ and Reverse 5′-ACG ATG CAA GAT GAA ACG GG-3′, glutaminase (GLA, human): Forward 5′-CGA TTT GTG GGG TGT GTC TG-3′ and Reverse 5′-CCA CTC GGC TCT TTT CCA AC-3′, glyceraldehyde-3-phosphate dehydrogenase (GAPDH, human): Forward 5′-TCA AGA AGG TGG TGA AGC AGG-3′ and Reverse 5′-TCA AAG GTG GAG GAG TGG GT-3′. GAPDH (mouse): Forward 5′-GAG ACA GAC AAG TAG ACC AA-3′ and Reverse 5′-CAG CAC CAA TAG TTG AAC AT-3′.

### Western blot analysis

Cultured cells were lysed in radioimmunoprecipitation assay (RIPA) buffer (Beyotime, China) containing a protease inhibitor cocktail with 1 mM phenylmethanesulfonyl fluoride (Beyotime, China). Total proteins were separated by sodium dodecyl sulfatepolyacrylamide gel electrophoresis (SDS-PAGE), and transferred onto a polyvinylidene difluoride (PVDF) membrane (Millipore Corporation, U.S.A.) before blocking with 5% skim milk in TBS-Tween for 2 h. Membranes were incubated with rabbit anti-α-smooth muscle actin (anti-α-SMA, Proteintech, China; 1:1000), rabbit anti-IDH2 (Sigma Aldrich, U.S.A.; 1:1000), or rabbit anti-β-actin (Proteintech, China; 1:1000) overnight at 4°C. Finally, membranes were incubated with horseradish peroxidase (HRP) conjugated anti-rabbit secondary antibody (Beyotime, China; 1:1000) at room temperature for 2 h; β-actin was used as a loading control. Positive bands were visualized with the Immobilon Western Chemiluminescent HRP Substrate kit (Millipore, U.S.A.). Images were taken using Amersham Imager 600 (AI600, GE, U.S.A.) and analyzed with ImageJ software.

### Statistical analysis

All data are presented as mean ± SD. Data were analyzed by one-way analysis of variance (ANOVA) using IBM SPSS statistics version 22 (IBM, U.S.A.). *P* < 0.05 was considered statistically significant.

## Results

### Intracellular α-ketoglutarate levels and IDH2 expression decreased with LX-2 HSC cell line activation

**Figure 1:**
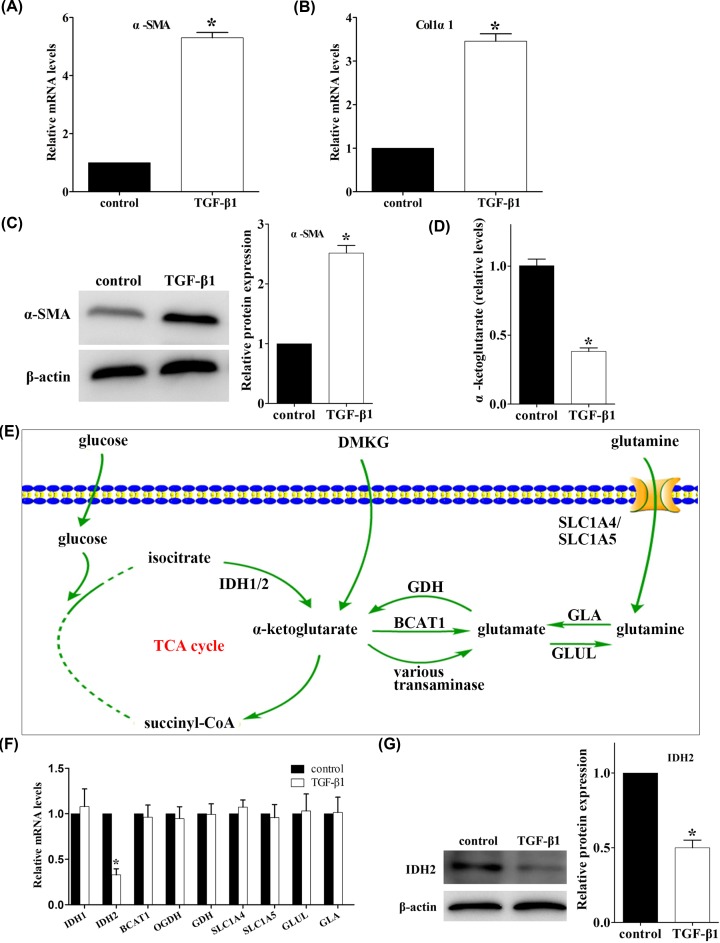
Intracellular α-ketoglutarate levels and IDH2 expression decreased with LX-2 cell activation (**A** and **B**) mRNA levels of α-SMA and collagen I α1 (Colα1) in LX-2 cells incubated with 5 ng/ml TGF-β1 for 24 h, using as GAPDH control (*n* = 3), *P < 0.05 vs. control group. (**C**) Western blot analysis of α-SMA expression from lysates of LX-2 cells incubated with 5 ng/ml TGF-β1 for 24 h, quantified as the ratio α-SMA/β-actin (*n* = 3), *P < 0.05 vs. control group. (**D**) Intracellular α-ketoglutarate levels in LX-2 cells incubated with 5 ng/ml TGF-β1 for 24 h (*n* = 3), *P < 0.05 vs. control group. (**E**) Schematic representation of the current paradigm of the α-ketoglutarate metabolic pathway in other cell types. Channel proteins (SLC1A4 and SLC1A5) and metabolic enzymes (IDH1, IDH2, BCAT1, OGDH, GDH, GLUL and GLA) are involved in the metabolism of α-ketoglutarate. (**F**) mRNA expression of IDH1, IDH2, BCAT1, OGDH, GDH, SLC1A4, SLC1A5, GLUL and GLA from lysates of LX-2 cells incubated with 5 ng/ml TGF-β1 for 24 h, using as GAPDH control (*n* = 3), *P < 0.05 vs. control group. (**G**) Western blot analysis of IDH2 expression from lysates of LX-2 cells incubated with 5 ng/ml TGF-β1 for 24 h, quantified as the ratio IDH2/β-actin (*n* = 3), *P < 0.05 vs. control group. IDH1, isocitrate dehydrogenase 1; IDH2, isocitrate dehydrogenase 2; BCAT1, branched-chain aminotransferase 1; α-KGDH, α-ketoglutarate dehydrogenase complex; OGDH, oxoglutarate dehydrogenase; GDH, glutamate dehydrogenase 1; SLC1A4, solute carrier family 1 (glutamate/neutral amino acid transporter), member 4; SLC1A5, solute carrier family 1 (glutamate/neutral amino acid transporter), member 5; GLUL, glutamate-ammonia ligase; GLA, glutaminase.

Using a well validated immortalized human HSC cell line (LX-2), we first detected the changes of intracellular α-ketoglutarate levels when HSC activation was induced by TGF-β1. A significant increase in the mRNA levels of the markers for HCSs activation α-SMA and Col1α1 was observed in TGF-β1 treated LX-2 cells (Figure 1A,B), as well as in protein levels of α-SMA (Figure 1C), while a significant reduction in intracellular α-ketoglutarate levels were detected (Figure 1D). We also measured mRNA levels of IDH1, IDH2, BCAT1, OGDH, GDH, SLC1A4, SLC1A5, GLUL and GLA, which are known to be involved in the metabolic processes of intracellular α-ketoglutarate (Figure 1E). The mRNA expression levels of IDH2 were significantly down-regulated in TGF-β1-treated LX-2 cells, while no differences for the expression of the other mRNAs were observed (Figure 1F). As we found that IDH2 mRNA was down-regulated in TGF-β1-treated LX-2 cells, we were interested if this translated to a subsequent reduction in protein expression levels. We demonstrated that protein expression of IDH2 was also significantly lower in TGF-β1-treated group than the controls (Figure 1G).

### Intracellular α-ketoglutarate level and IDH2 expression decreased during primary mouse HSC activation

**Figure 2:**
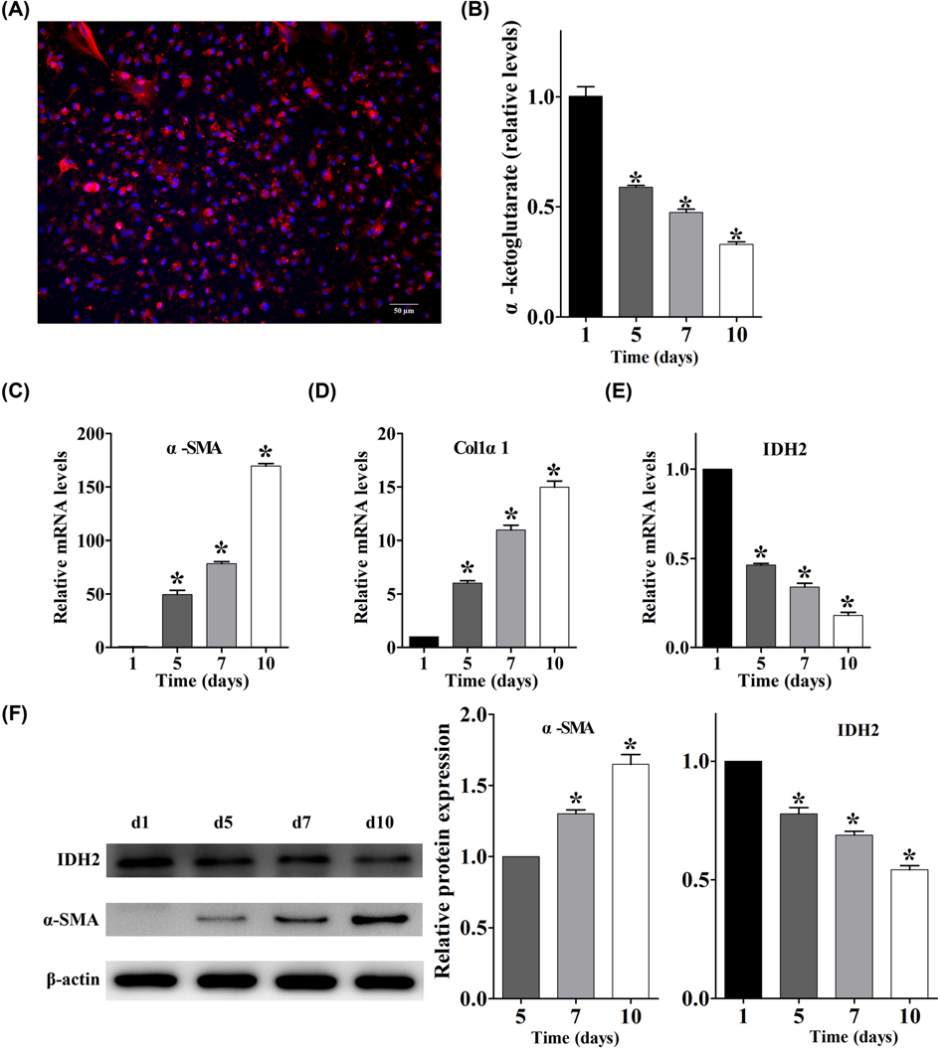
Intracellular α-ketoglutarate levels and IDH2 expression decreased with primary mouse HSC activation (**A**) Representative images of primary mouse HSCs (day 1) after immunofluorescent staining of desmin; scale bar = 50 μm. (**B**) Intracellular α-ketoglutarate levels post-activation in primary mouse HSCs at day 1, 5, 7 and 10 *in vitro* (*n* = 3), *P < 0.05 vs. day 1 group. (**C**–**E**) mRNA levels of α-SMA, Colα1 and IDH2 in primary mouse HSCs at different times of *in vitro* activation, using as GAPDH control (*n* = 3), *P < 0.05 vs. day 1 group. (**F**) Western blot analysis of α-SMA and IDH2 expression from lysates of primary mouse HSCs at different times of *in vitro* activation, using β-actin as control (*n* = 3), *P < 0.05 vs. day 5 group for α-SMA, *P < 0.05 vs. day 1 group for IDH2.

To verify the observations in LX-2 cells, we isolated primary HSCs from mouse livers (Figure 2A). Freshly isolated cells were considered quiescent. Without adding TGF-β1, the activation levels continued to rise with each subsequent day of culture as the primary HSC become fully differentiated to the myofibroblast phenotype [18]. A detailed time course for primary HSC activation (day 1 to 10) revealed that the intracellular α-ketoglutarate levels were significantly decreased (Figure 2B) with a significant increase in the mRNA levels of α-SMA and Col1α1 (Figure 2C,D), while a significant reduction in the mRNA levels of IDH2 were detected (Figure 2E). Western blot analysis confirmed a significant reduction in IDH2 protein expression levels upon primary HSC activation (Figure 2F).

### Knockdown of IDH2 promotes HSC activation that can be reversed by an α-ketoglutarate analogue

**Figure 3:**
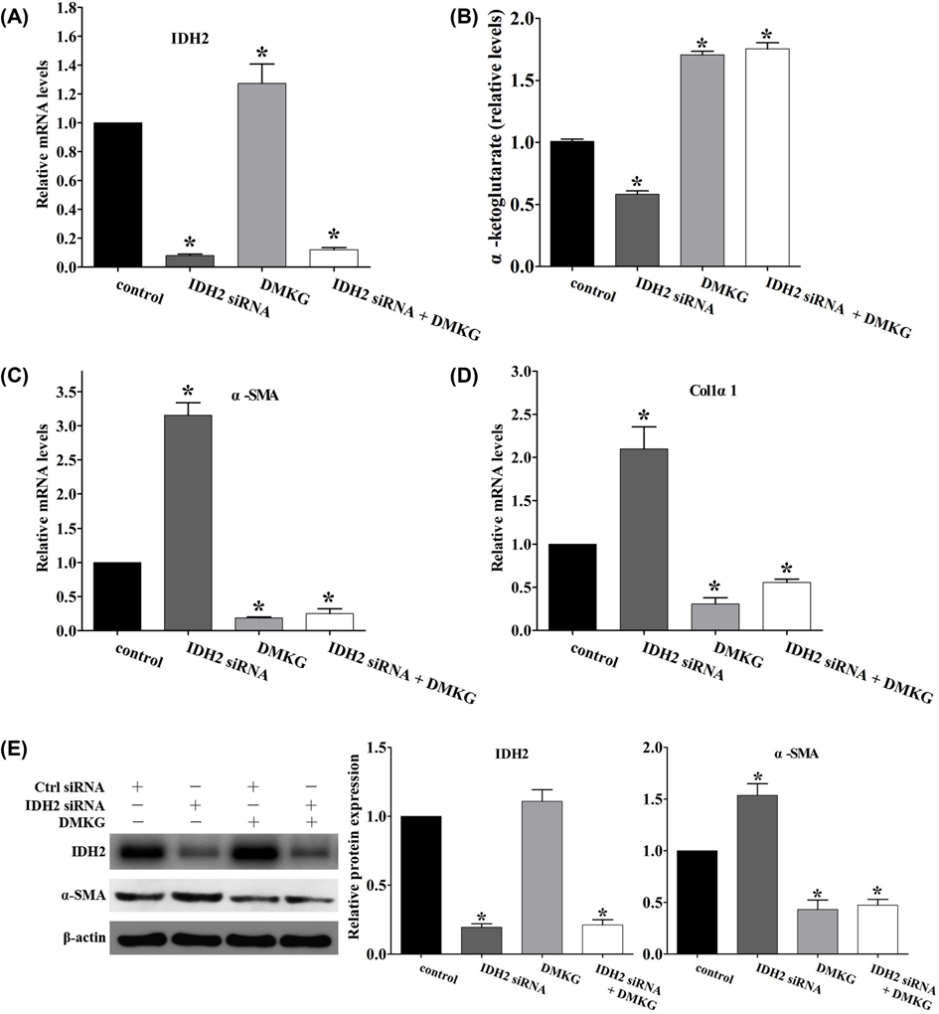
Knockdown of IDH2 promotes LX-2 cell activation that was reversed by DMKG LX-2 cells were transfected with IDH2 siRNA or control siRNA for 48 h. The cells were then left untreated or treated with 4mM DMKG for 24 h. (**A**) mRNA levels of IDH2 in LX-2 cells, using as GAPDH control (*n* = 3), *P < 0.05 vs. control siRNA transfected group. (**B**) Intracellular α-ketoglutarate levels (*n* = 3), *P < 0.05 vs. control siRNA transfected group. (**C** and** D**) mRNA levels of α-SMA and Colα1 in LX-2 cells, using as GAPDH control (*n* = 3), *P < 0.05 vs. control siRNA transfected group. (**E**) Western blot analysis was used to determine IDH2 and α-SMA expression of the cells, using β-actin as a control (*n* = 3), *P < 0.05 vs. control siRNA transfected group.

To investigate the effect of IDH2 on intracellular α-ketoglutarate levels and HSC activation, we used siRNA to knockdown the expression of IDH2 in LX-2 cells (Figure 3A). Knockdown of IDH2 significantly decreased intracellular α-ketoglutarate levels (Figure 3B), while the mRNA levels of α-SMA and Col1α1 (Figure 3C,D) and the protein expression of α-SMA (Figure 3E) were enhanced in IDH2-siRNA treated cells. Dimethyl α-ketoglutarate (DMKG) is a membrane-permeable ester of α-ketoglutarate [19,20]. With the addition of DMKG, enhanced levels of intracellular α-ketoglutarate were detected (Figure 3B), while no significant difference in the mRNA levels of α-SMA or Col1α1 (Figure 3C,D) or the protein expression of α-SMA (Figure 3E) between IDH2-siRNA + DMKG and control siRNA + DMKG group was observed. These results indicate that inhibition of IDH2 promotes HSC activation, which can be reversed by the addition of α-ketoglutarate analogue.

### α-Ketoglutarate regulates HSC activation independent of TGF-β1 stimulation

**Figure 4:**
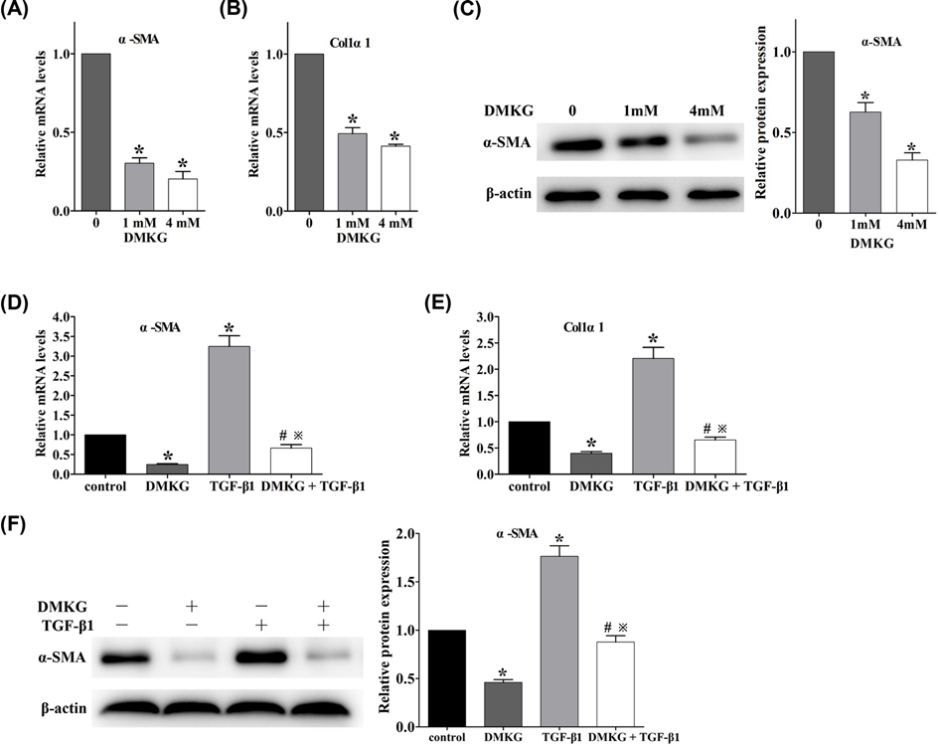
DMKG reduced primary mouse HSC activation independent of TGF-β1 stimulation (**A** and **B**) mRNA levels of α-SMA and Colα1 in primary mouse HSCs (day 7) left untreated or treated with DMKG (1 and 4 mM) for 24 h, using as GAPDH control (*n* = 3), *P < 0.05 vs. control group. (**C**) Western blot analysis of α-SMA expression from lysates of primary mouse HSCs (day 7) left untreated or treated with DMKG (1 and 4 mM) for 24 h, quantified as the ratio α-SMA/β-actin (*n* = 3), *P < 0.05 vs. control group. (**D** and** E**) mRNA levels of α-SMA and Colα1 in primary mouse HSCs (day 7) incubated with control media or DMKG (4 mM) in the presence or absence of TGF-β1 (5 ng/ml) for 24 h, using as GAPDH control (*n* = 3), *P < 0.05 vs. control group, ^#^*P* < 0.05 vs. DMKG-treated group, ^※^*P* < 0.05 vs. TGF-β1-treated group. (**F**) Western blot analysis of α-SMA expression from lysates of primary mouse HSCs (day 7) incubated with control media or DMKG (4 mM) in the presence or absence of TGF-β1 (5 ng/ml) for 24 h, quantified as the ratio α-SMA/β-actin (*n* = 3), *P < 0.05 vs. control group, ^#^*P* < 0.05 vs. DMKG-treated group, ^※^*P* < 0.05 vs. TGF-β1-treated group.

One of our previous study has confirmed that DMKG could reduce the activation of activated HSCs [15]. In the present study, we will further verify the conclusions drawn from previous cell lines using primary HSCs. Without TGF-β1 stimulation, the activated phenotype of HSC can be obtained in primary cells 7 days after culture *in vitro* [18]. Treatment of primary mouse HSCs (day 7) with 1- or 4mM-DMKG significantly decreased the mRNA levels of α-SMA and Col1α1 (Figure 4A,B), as well as protein levels of α-SMA (Figure 4C). Moreover, in the present study, we will verify whether DMKG and TGF-β1 can play their own independent regulatory role in HSC activation by detecting the mRNA content of α-SMA and Col1a1 (Figure 4D,E), the indicators of HSC activation, and protein expression of α-SMA (Figure 4F). We found that TGF-β1 could still promote HSC activation with DMKG addition (group DMKG+TGF-β1 vs. group DMKG), that is, there was no TGF-β1–α-ketoglutarate activation axis. With TGF-β1 addition, DMKG could still inhibit HSC activation (group DMKG+TGF-β1 vs. group TGF-β1), that is, there was no α-ketoglutarate–TGF-β1 activation axis. The results indicate that DMKG can regulate HSC activation in a mechanism independent TGF-β1.

## Discussion

The major finding of the present study is the identification of the mechanism and role of intracellular α-ketoglutarate reduction in the activation of HSCs. First, intracellular α-ketoglutarate was significantly reduced in an HSC cell line, LX-2, after stimulation with TGF-β1 (Figure 1A–D). Second, IDH2 expression was significantly down-regulated with the consequent reduction of intracellular α-ketoglutarate (Figure 1E–G). Third, the results mentioned above were replicated with primary mouse HSC activation (Figure 2). Additionally, siRNA knockdown of IDH2 *in vitro* promoted the activation of HSCs, which was reversed by supplementation of the exogenous α-ketoglutarate analogue, DMKG (Figure 3) [19,20]. Finally, regulation of intracellular α-ketoglutarate on activation of HSCs was through a TGF-β1 independent pathway (Figure 4). Collectively, these data demonstrated that decrease in IDH2 expression limits the production of α-ketoglutarate during HSC activation and the reduction of intracellular α-ketoglutarate in turn promotes the activation of HSCs through a TGF-β1 independent pathway.

Our previous study revealed that increase in intracellular α-ketoglutarate by DMKG inhibited the activation of HSCs [15,16], while our present study showed that decrease in intracellular α-ketoglutarate by IDH2-siRNA promoted HSC activation. Our studies are the first to show that intracellular α-ketoglutarate can regulate HSC activation. Our conclusion is consistent with the data from the literature. Araujo et al*.* showed that exposure to 3,4-Methylenedioxymethamphetamine (MDMA) induces a significant reduction in intracellular α-ketoglutarate in hepatocytes [21], while Varela-Rey et al*.* demonstrated that MDMA could directly promote the activation of HSCs *in vitro* [22]. Interestingly, a recent study by Tischler et al*.* showed that high levels of intracellular α-ketoglutarate inhibited the differentiation of embryonic stem cells and indicated that α-ketoglutarate also could stabilize transitory cellular states in other contexts [9]. This potentially may provide a universal tool for capturing and expanding short-lived cell states *in vitro* through metabolic modulation. HSC activation is a progress that shifts HSCs from a quiescent state to a myofibroblast-phenotype and the quiescent HSC is an unstable and transient cellular state during HSC activation [1].

Although it has been reported some channel proteins and metabolic enzymes including IDH1, IDH2, BCAT1, OGDH, GDH, SLC1A4, SLC1A5, GLUL, and GLA were involved in the metabolism of α-ketoglutarate in many cell types [9,23–31], the mechanism of intracellular α-ketoglutarate regulation during HSC activation has not been previously reported. The main metabolic sources of intracellular α-ketoglutarate including glucose and glutamine were kept sufficient in the cell culture medium in our research [32,33], while the expression of channel proteins and metabolic enzymes mentioned above were investigated during HSC activation. We found the regulation of intracellular α-ketoglutarate was dependent on IDH2, an enzyme that converts citrate to α-ketoglutarate. Similarly, Tischler et al*.* reported that the reduction or depletion of intracellular α-ketoglutarate during embryonic stem cell differentiation was by limiting IDH2-mediated conversion of citrate into α-ketoglutarate [9]. Moreover, Liu et al*.* demonstrated that melatonin elevated α-ketoglutarate levels by regulating IDH2 in adipocytes while Das et al*.* and Shajari et al*.* also demonstrated that melatonin could suppress the activation of HSCs [31,34,35]. Although our present study showed that decrease in intracellular α-ketoglutarate by IDH2-siRNA promoted HSC activation, further study of expression of IDH2 gene to restore the reduced α-ketoglutarate in HSC activation is needed.

Autophagy fuels the activation of HSCs [36], while glutaminolysis might fuel anapleurosis to meet the elevated energy demands for the myofibroblast phenotype [37]. Our previous research demonstrated that α-ketoglutarate, the end-product of glutaminolysis, reduced the energy production and activation of HSCs through inhibition of autophagy in HSCs [15]. Consistent with our study, a study by Marino et al*.* demonstrated that α-ketoglutarate could inhibit the fibrogenisis of cardiomyocytes [19]. Moreover, Chin et al*.* demonstrated that α-ketoglutarate could suppress energy production by directly inhibiting ATP synthase [38]. Therefore, we conclude that α-ketoglutarate inhibits energy production in HSCs. Further investigation on α-ketoglutarate and bioenergetic is warranted. Although TGF-β1 is the most potent pro-fibrogenic cytokine [8], we showed that the regulation of α-ketoglutarate on the activation of HSCs might be TGF-β1 independent. Our studies elucidate a new approach for blocking the progression of liver fibrosis through the regulation of α-ketoglutarate and IDH2 in HSCs; however, further studies are still required.

## Abbreviations

HSC: hepatic stellate cell
IDH2: isocitrate dehydrogenase 2
RIPA: radioimmunoprecipitation assay
TGF-β1: transforming growth factor-β1
